# Small Choroidal Melanoma with Monosomy 3

**DOI:** 10.4103/0974-9233.65487

**Published:** 2010

**Authors:** Fariba Ghassemi, Carol L. Shields, Miguel A. Materin, Jerry A. Shields

**Affiliations:** Department of Ocular Oncology Service, Wills Eye Institute, Philadelphia, PA, USA

**Keywords:** Choroidal Melanoma, Chromosome 3, Eye, Metastases, Monosomy, Small Choroidal Melanoma

## Abstract

**Purpose:**

To report a patient with small juxtapapillary choroidal melanoma with chromosome 3 monosomy treated with I^125^ plaque and transpupillary thermotherapy (TTT). A 64-year-old Caucasian male presented with painless blurred vision of the left eye. Ocular examination disclosed a small juxtapapillary choroidal melanocytic tumor with overlying subretinal fluid and orange pigment. Ultrasound showed an elevated choroidal mass of 2 mm thickness with low reflectivity on A-scan and hollowness on B scan, consistent with a small choroidal melanoma. The patient was treated with plaque I^125^ radiotherapy combined with one session of TTT. Genetic testing of the tumor cells obtained by fine needle aspiration biopsy showed chromosome 3 monosomy. At 1 year after treatment, the tumor was regressed with resolution of subretinal fluid and 20/40 visual acuity. A small choroidal melanoma can manifest monosomy of chromosome 3, a known predictive factor for the development of systemic metastasis.

## INTRODUCTION

The management of small choroidal melanocytic tumors has traditionally been cautious observation until documented growth. In 1995, Shields *et al*. reported risk factors predictive of growth and metastasis of small melanocytic tumors in an effort to improve detection of small choroidal melanoma.^1^ Choroidal melanocytic tumors that displayed three or more factors had 50% or greater risk for growth. Those patients with all of the risk factors for metastases (blurred vision, thickness ≥ 2.0 mm, abutting the optic disc, and with documented growth) showed 154 times greater risk for metastasis than those patients without them. In this study, early treatment without waiting for growth was advocated, with the hope that this strategy might improve patient survival.^1^

More recently, genetic testing has added an important dimension to the management of patients with choroidal melanoma.^2^,^3^ Monosomy of chromosome 3 is believed to be the most important genetic abnormality predictive of systemic metastasis and has been found in approximately 57% of patients with medium and large melanoma at enucleation.^2^ We report a very small choroidal melanoma with monosomy 3 abnormality.

## CASE REPORT

**Figure 1:**
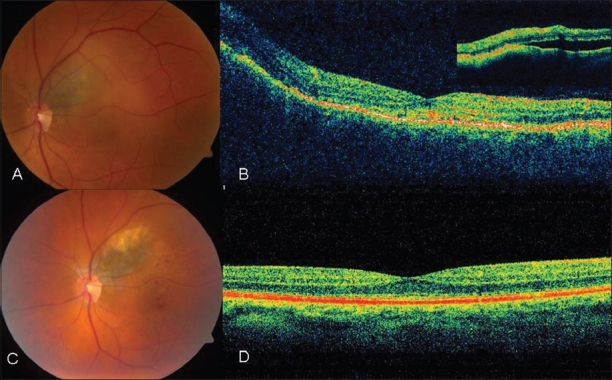
Small juxtapapillary choroidal melanoma with monosomy of chromosome 3. (A) Fundus exam showed a small juxtapapillary choroidal melanoma that measured 2 mm in thickness. Overlying orange pigment and subretinal fluid were visible. (B) Horizontal optical coherence tomography through the foveal area showed subfoveal debris and the elevated choroidal mass. The inset shows subretinal fluid over the tumor. (C) One year after plaque radiotherapy and thermotherapy, the melanoma showed regression to 1.2 mm thickness and with fine overlying intraretinal hemorrhages consistent with mild radiation retinopathy. (D) One year after treatment, the horizontal optical coherence tomography showed no subretinal fluid and flattened choroidal mass

A 64-year-old Caucasian male developed blurred vision of the left eye (OS) over 4 months. A choroidal nevus was diagnosed 15 years earlier in the left eye, but no photographs were taken. Visual acuity was 20/20 in the right (OD) and 20/40 in the left eye (OS). The right fundus was unremarkable. The left fundus revealed a small juxtapapillary choroidal melanocytic tumor with overlying subretinal fluid and orange pigment without any halo and drusen [Figure 1A]. The mass measured 5 × 4 mm^2^ in basal diameter.

Ultrasonography showed the lesion to be 2 mm thick, with low-to-medium internal reflectivity. Optical coherence tomography revealed subretinal fluid without retinal degenerative changes [Figure 1B]. There was hyperautofluorescence of orange pigment on the tumor surface. A diagnosis of small choroidal melanoma was rendered and treatment with a notched I^125^ plaque combined with one session of transpupillary thermotherapy (TTT) was provided. At the time of plaque placement, fine needle aspiration biopsy (FNAB) was performed and genetic testing revealed monosomy 3. At 1 year after treatment, the visual acuity was 20/40 and the tumor remains regressed and flat with no subretinal fluid [Figure 1C, D].

## CONCLUSIONS

In almost all kinds of cancers, the detection of early lesions and their appropriate management are still the most efficient ways of improving the patient’s prognosis.^4^ In dermatology, the mnemonic *ABCD* (Asymmetric, notched Borders, Color variability, Diameter ≥ 6 mm) has been found to tremendously aid in the identification of early cutaneous melanoma. Cutaneous melanoma is currently detected at a mean thickness of 0.76 mm. The survival worsens on a continuum with increasing thickness.^4^

The mnemonic TFSOM UHH D (to find small ocular melanoma using helpful hints daily) representing thickness >2 mm, subretinal fluid, symptoms, orange pigment, margin < 3 mm from disc and ultrasonographic hollowness, absence of Halo, and Drusen absence, is useful in identifying small choroidal melanocytic lesions with growth potential.^5^ Our patient displayed eight factors with a hazard ratio (HR) of 31 for growth compared to a lesion without those risk factors.^5^

Recently, chromosome 3 abnormality in uveal melanoma has been found to portend poor life prognosis.^2^ Shields *et al*. found that 26% of 61 small choroidal melanomas harbored monosomy 3; however, longer follow-up and further chromosomal investigation are needed to understand the prognostic implications.^3^ In conclusion, genetic testing of small choroidal melanoma using FNAB is feasible and, despite their small size, these tumors can have chromosomal changes that portend a worse prognosis.
